# Complete Genome Sequence of the Oral Spirochete Bacterium *Treponema putidum* Strain OMZ 758^T^ (ATCC 700334^T^)

**DOI:** 10.1128/genomeA.01076-14

**Published:** 2014-10-23

**Authors:** Donnabella C. Lacap-Bugler, Jingwei Jiang, Yong-Biao Huo, Yuki Chan, Frederick C. Leung, Rory M. Watt

**Affiliations:** a Oral Biosciences, Faculty of Dentistry, The University of Hong Kong, Pok Fu Lam, Hong Kong SAR, China; b School of Biological Sciences, Faculty of Science, The University of Hong Kong, Pok Fu Lam, Hong Kong SAR, China; c Bioinformatics Centre, Nanjing Agricultural University, Jiangsu, China

## Abstract

The oral spirochete bacterium *Treponema putidum* inhabits human periodontal niches. The complete genome sequence of the OMZ 758^T^ (ATCC 700334^T^) strain of this species was determined, revealing a 2,796,913-bp chromosome, with a G+C content of 37.30% and a single plasmid (pTPu1; 3,649 bp) identical to pTS1 from *Treponema denticola*.

## GENOME ANNOUNCEMENT

More than 75 species or phylotypes of oral spirochetes inhabit the oral cavity (1, 2). All taxa belong to the genus *Treponema* and are classified into 10 phylogroups (3). Phylogroup 2 contains two formally described species, *Treponema denticola* (4) and *Treponema putidum* (5), which share ca. 98.5% 16S rRNA gene sequence homology. The genome sequence of *T. denticola* ATCC 35405^T^ was published in 2004 (6). A large number of clinical studies have associated *T. denticola* with human periodontal diseases (reviewed in references 7–9). Unlike *T. denticola*, the ecology, distributions, and clinical disease associations of *T. putidum* are poorly understood. Here, we report the complete genome of the type strain of *T. putidum* (OMZ 758^T^, ATCC 700334^T^), which was originally isolated from a Swiss periodontitis patient (5).

The OMZ 758^T^ strain was obtained directly from the original isolator and depositor (C. Wyss, University of Zurich) and was cultured anaerobically in supplemented tryptone-yeast extract-gelatin-volatile fatty acids-serum (TYGVS) medium, as previously described (10). Genomic DNA was extracted from a pure culture (QIAamp DNA mini extraction kit; Qiagen, Germany) and was sequenced using a 454 Life Sciences GS Junior platform at the Nanjing Agricultural University Bioinformatics Centre, Jiangsu, China. An initial shotgun library generated 245,625 reads, which was followed by an 8-kb span paired-end library yielding 119,479 reads, generating sequence data with 54× coverage. Using 454 Newbler version 2.7, the *de novo* assembly resulted in 40 large contigs, with an *N*_50_ contig size of 264,778 bp in two scaffolds. The single gap was closed by PCR and Sanger sequencing.

The *T. putidum* OMZ 758^T^ chromosome is 2,796,913 bp, with a G+C content of 37.30%. Annotation was performed automatically using the PGAAP pipeline implemented in NCBI, following the best-placed reference protein set (GeneMarkS+ version 2.7). The genome contains 2,448 genes, 176 pseudogenes, 42 tRNAs, and two copies of an rRNA cluster (5S, 16S, and 23S). There is also a single plasmid of 3,649 bp (pTPu1), with a G+C content of 34.17%, which shares 100% sequence similarity with *T. denticola* plasmid pTS1 (accession no. AF112856) (11). The genome encodes homologues of virulence factors previously identified within *T. denticola* (7–9). These include the major surface protein (MSP) (locus tag JO40_03880) involved in cellular adhesion processes (12), factor H binding protein (FhbB, FHL-1) (locus tag JO40_03575), implicated in evading complement-mediated killing (13), and cystalysin (hemolysin [Hly]) (locus tag JO40_09085), involved in volatile sulfur compound production and erythrolysis (14).

The annotated full-genome sequence for *T. putidum* OMZ 758^T^ (ATCC 700334^T^) reported here represents the first strain for this species. This significantly broadens the genomic data available for studying the biology, physiology, and phylogeny of oral treponemes associated with periodontal diseases. It will also facilitate efforts to systematically analyze genetically related species of treponemes that inhabit oral and nonoral niches in animals, many of which are implicated in polymicrobial tissue-destructive diseases (15, 16).

### Nucleotide sequence accession numbers.

This complete genome sequence has been deposited in GenBank under the accession no. CP009228. The pTPu1 plasmid sequence has been deposited under the accession no. CP009229.
